# MicroRNA-381-3p signatures as a diagnostic marker in patients with sepsis and modulates sepsis-steered cardiac damage and inflammation by binding HMGB1

**DOI:** 10.1080/21655979.2021.2006967

**Published:** 2021-12-11

**Authors:** Jian Liu, Yadong Yang, Rong Lu, Qin Liu, Shukun Hong, Zhaolong Zhang, Guoxin Hu

**Affiliations:** a Department of Intensive Medicine, Shengli Oilfield Central Hospital, Dongying, China; b Department of Emergency, Shengli Oilfield Central Hospital, Dongying, China; c Department of Laboratory, Shengli Oilfield Central Hospital, Dongying, China

**Keywords:** MiR-381-3p, viability, sepsis, inflammation, HMGB1, myocardial dysfunction

## Abstract

Immune response imbalance and cardiac dysfunction caused by sepsis are the main reasons for death in sepsis. This study aimed to confirm the expression and diagnostic possibility of microRNA-381-3p (miR-381-3p) and its mechanism in sepsis. Quantitative real-time PCR (qRT-PCR) and receiver operating characteristic (ROC) were used to reveal the levels and clinical significance of miR-381-3p. Pearson correlation was conducted to provide the correlations between miR-381-3p and several indexes of sepsis. The H9c2 cell models were constructed by lipopolysaccharide (LPS), and cecal ligation and puncture (CLP) was applied to establish the Sprague-Dawley (SD) rat models. Cell Counting Kit-8 (CCK-8) and flow cytometry were the methods to detect the cell viability and death rate of H9c2. Enzyme-linked immunosorbent assay (ELISA) was performed to evaluate the concentration of inflammatory cytokines. The target gene of miR-381-3p was validated via the luciferase report system. The low expression of miR-381-3p was found in the serum of patients with sepsis. The lessened miR-381-3p could be a marker in the discrimination of sepsis patients. Overexpression of miR-381-3p could repress the mRNA expression of HMGB1, inhibit the cell apoptosis and inflammatory response, and motivate the viability of sepsis cells. At the same time, enhanced miR-381-3p promoted the inhibition of inflammation and cardiac dysfunction in the rat model of sepsis. Collectively, reduced levels of serum miR-381-3p can be used as an index to detect sepsis patients. MiR-381-3p restored the inflammatory response and myocardial dysfunction caused by sepsis via HMGB1.

## Introduction

Sepsis is one common disease faced by doctors in critical medicine and its pathophysiological mechanism is relatively complicated [1]. At present, it is considered as a risk of death caused by organ dysfunction and waterfall inflammation [2,3]. If the disease worsens, it can further evolve into systemic disorders or even worsen numerous organ failures, and the heart is one of the main damaged target organs in the process of sepsis [4]. If not monitored and treated in time, it will affect the hemodynamics of patients, lead to further deterioration of cardiac function and aggravate the overall condition [5]. Cardiac markers commonly used in the clinic, among which cardiac troponin I (cTnI) and creatine kinase isoenzyme (CK-MB) are diffusely used markers of myocardial injury [6,7]. But they are affected by the mechanism of release and clearance, disease status, and complications involving organs, so there are limitations in the diagnosis of myocardial injury in sepsis. And it can not be used as a therapeutic target for myocardial injury, and it has limited effect on improving the prognosis of patients. Therefore, it is particularly important to find biomarkers with good specificity and sensitivity.

Recent studies have provided that the expression profiles of circulating microRNA (miRNA) may change in a variety of pathological situations, such as infection, cardiac function impairment, and sepsis [8]. In the future, differentially expressed miRNA can be used as early sepsis biomarkers and have the potential for early diagnosis of sepsis [9,10]. In terms of easy handling conditions, sensitivity and specificity, the serum miRNA expression is superior to traditional biological markers in the assessment of sepsis [11]. More importantly, the alternation of miR-381-3p levels and the regulatory role of miR-381-3p have been reported in many human immune diseases and heart-related diseases. For example, in polymyositis, the expression levels of miR-381-3p are lessened in patients with polymyositis, and miR-381-3p reduces inflammation and macrophage infiltration in polymyositis by down-regulating HMGB1 [12]. In coronary heart disease, the expression of miR-381-3p is significantly diminished compared with the normal control, and its overexpression can significantly inhibit the release of inflammatory factors in human umbilical vein endothelial cells [13]. This study indicated miR-381-3p might be a protective role in inflammatory dysfunction. MiR-381-3p expression may show different trends due to different disease types, and thus plays different biological roles in various diseases. But there are few reports on miR-381-3p in sepsis, and people have a limited understanding of the function of miR-381-3p in sepsis.

Therefore, we hypothesized that the expression of miR-381-3p might be associated with sepsis. This study aimed to find an alternative biomarker in sepsis. This subject proposed to verify the expression of miR-381-3p in sepsis, deeply analyze its clinical significance and biological function in sepsis, and explore relevant molecular mechanisms.

## Materials and Methods

### Included patients

A total of 207 volunteers (102 sepsis patients and 105 healthy participants) from Shengli Oilfield Central Hospital were included in this investigation. Admission criteria of the sepsis group were based on the definition and diagnostic criteria of sepsis in 2016 [14]. Exclusion criteria were as follows: (1) pregnant women; (2) age < 18 years old; (3) patients with autoimmune diseases; (4) patients with previous chest trauma or cardiac surgery; (5) patients with organic heart disease (such as coronary heart disease, cardiomyopathy, and acute cor pulmonale, etc.) and congenital heart disease; (6) patients with chronic renal insufficiency and neuromuscular diseases; (7) tumors. Blood samples of volunteers were collected separately, and serum samples were separated by centrifugation and stored at −80°C for later use. The experimental design and scheme have been approved by the Ethics Committee of Shengli Oilfield Central Hospital and obtained the written and informed consent of all participants. Acute physiology and chronic health evaluation II (APACHE II) and sequential organ failure assessment (SOFA) of sepsis patients were calculated. Record in detail the general data such as age, sex, height, weight, and library indexes of patients and healthy persons. Blood samples were obtained from untreated patients with sepsis within 24 hours of submission.

### H9c2 cell models establishment and transfection

Cardiomyocytes H9c2 were purchased from Procell (Wuhan, China). All cells were cultured in Dulbecco’s modified Eagle’s medium (DMEM) with 10% FBS and placed in a 37°C incubator containing 5% CO_2_. The septic cell models were established by adding 10 μg/ml lipopolysaccharide (LPS, Solarbio, Beijing, China) to DMEM and culturing for 12 hours [15]. The passage number of H9c2 cells used in our experiments was 10–25.

The synthesized miR-381-3p mimics, miR-381-3p inhibitors, and miR-381-3p-negative control (NC) were obtained from Genepharm (Shanghai, China). These oligonucleotides dissolved and diluted with diethylpyrocarbonate-treated water, and stored at −20°C for subsequent use. H9c2 cells were inoculated into 6-well plates at a cell density of 1 × 10^5^ cells/well. The target sequences (20 nM) were transfected by Lipofectamine™ 3000 at 37°C for 48 hours.

### Evaluation of cell viability and apoptosis

The cell viability was detected via the CCK-8 from GLPBIO (Shanghai, China) based on the suggested procedure. The values at 450 nm expressed the viability of H9c2 cells. Besides, the apoptotic cells influenced by CLP were accessed by the FITC-Annexin V Apoptosis Detection kit (BD Biosciences, San Jose, USA) per the recommended suggestions. The apoptotic value was equal to the sum of the late and early apoptosis values.

### Sepsis animal grouping and parameters’ detection

The Sprague-Dawley (SD) model of sepsis was constructed with cecal ligation and puncture (CLP) [16]. Male SD rats of 8–10 weeks old and weighing 250~300 g of clean grade were purchased from JKbiot (Nanjing, China). The rats were randomly divided into the sham group, CLP, CLP + agomir-NC, and CLP + miR-381-3p agomir groups. For rats in the CLP + miR-381-3p agomir and CLP + agomir-NC groups, miR-381-3p agomir and their controls were used for intravenous injection of rat tails separately. These sequence reagents were purchased from Genepharm (Shanghai, China). Rats in the sham group and CLP model group were then given an equal volume of sterile normal saline. Five rats were randomly included in each group. The blood samples were collected after 24 hours of treatment. All animal experiments were conducted following the Guide for the Care and Use of Laboratory Animals and approved by the ethics committee Shengli Oilfield Central Hospital.

### Cardiac function analysis

SD models were anesthetized by intraperitoneal injection of 0.2 ml/100 g of 1.5% sodium pentobarbital injection at 48 hours of surgery. After anesthesia, rats were subjected to tracheostomy and mechanical ventilation. The PowerLab data analysis and processing system were connected, and the catheter was inserted into the left ventricle to measure the left ventricular systolic pressure (LVSP) left ventricular end-diastolic pressure (LVEDP), maximum rate of increase of left ventricular internal pressure (+dp/dtmax), maximum rate of decrease of left ventricular internal pressure (-dp/dtmax), the left ventricular mass (LVM), and left ventricular wall thickness (LVWT) [16,17]. After that, the blood was extracted for the following tests.

### RNA extraction and quantitative real-time PCR (qRT-PCR)

The total RNA from serum and cells was collected with Trizol LS reagent, and the purity of the resulting RNA was measured using NanoDrop 2000. RNA was reverse transcribed into cDNA using the AMV reverse transcription kit following the instructions for use. The synthesized cDNA was used as a template, and qPCR was used to measure the relative expression level of miR-381-3p. For HMGB1, the cDNA was obtained using a universal RT-PCR kit (Solarbio, Beijing, China). The primers were as follows: human miR-381-3p forward, 5ʹ-GGAGCCTATACAAGGGCAAGC-3ʹ, reverse 5ʹ-GCGAGCACAGAATAAATACGACTCACTA-3ʹ; rat miR-381-3p forward, 5ʹ-AGTCTATACAAGGGCAAGCTCTC-3ʹ, reverse, 5ʹ-ATCCATGACAGATCCCTACCG-3ʹ; human U6 forward, 5ʹ-CTCGCTTCGGCAGCACA-3ʹ, reverse, 5ʹ-AACGCTTCACGAATTTGCGT-3ʹ; rat U6 forward 5ʹ-GCTTCGGCAGCACATATACTAAAAT-3ʹ, reverse 5ʹ-CGCTTCACGAATTTGCGTGTCAT-3ʹ; HMGB1 forward 5ʹ-TATGGCAAAAGCGGACAAGG-3ʹ, reverse, 5ʹ-CTTCGCAACATCACCAATGGA-3ʹ; and GAPDH forward, 5ʹ-ATCAGCAATGCCTCCTGCAC-3ʹ, reverse, 5ʹ-CGTCAAAGGTGGAGGAGTGG-3ʹ. The reaction was performed using the SYBR Green PCR reaction mixture and ABI 7500 real-time PCR system. U6 was used as the internal reference for miR-381-3p and GAPDH was the endogenous reference for HMGB1. The relative expression of miR-381-3p was calculated using the 2^−ΔΔCt^ method.

### Enzyme-linked immunosorbent assay (ELISA)

The levels of inflammation indicators, cTnI, and CK-MB both in the serum samples of SD rats and supernatant of H9c2 cells were tested by the ELISA kits on the basis of respective specifications.

### Luciferase activity assay

The HMGB1 sequence containing binding sites and its mutation sequence were cloned into pcDNA3.1 vectors. Then, the vectors carrying HMGB1-mutation or HMGB1-wide were transfected into cells with miR-381-3p mimics, inhibitors, and controls. The luciferase detection was used by Luciferase Reporter Assay Kit (AmyJet Scientific, Wuhan, China).

### Statistical analysis

Using the SPSS and GraphPad statistical software, all the measurement data was represented by the statistical x ± s. Chi-square test, Mann-Whitney U-test, and T-test were used to compare the differences between the sepsis group and the control group. One-way ANOVA was used for comparison among groups. According to the expression level of miR-381-3p in the serum of patients and healthy volunteers, the prognostic ability of miR-381-3p was analyzed by the optimal cutoff value and the area under the curve (AUC) of the receiver operating characteristic (ROC). Pearson correlation analysis was used to analyze the correlation between variables. *P* < 0.05 means statistically significant. Each experiment was repeated five times.

## Results

In the present investigation, we aimed to assess the influence of miR-381-3p in sepsis. For this purpose, we examined the expression of miR-381-3p in patients with sepsis and evaluated the prognostic role of miR-371-3p in sepsis. In addition, the impacts of miR-381-3p on LPS-injured H9c2 cardiomyocytes and CLP-treated rats. Moreover, the target gene of miR-381-3p was clarified by luciferase reporter assay.

### Comparison of observation indexes between observation group and control group

There were no obvious differences in sex distribution, age, and body mass index between the 102 sepsis patients and the 105 control individuals (Table 1, *P* > 0. 05). Compared with the control group, the procalcitonin (PCT), white blood cell (WBC), C-reactive protein (CRP), and serum creatinine (Scr) in the observation group were significantly higher than those in the healthy group (Table 1, *P* < 0.001). The albumin concentration in sepsis patients was lower than that in the control individuals (Table 1, *P* < 0.001). Moreover, the SOFA and APACHE II scores were 5.02 ± 1.34 and 12.02 ± 5.58 separately.

**Table 1.** Comparison of the baseline data between the two groups

Parameters	Control (n = 105)	Sepsis (n = 102)	*P* value
Age (year)	55.93 ± 14.64	56.85 ± 12.69	0.630
BMI (kg/m^2^)	23.21 ± 2.94	22.94 ± 3.11	0.554
Gender (male/female)	53/52	53/49	0.831
PCT (ng/mL)	0.11 ± 0.04	7.72 ± 2.88	<0.001
WBC (×10^9^/L)	6.25 ± 1.39	15.51 ± 3.56	<0.001
CRP (mg/L)	6.38 ± 1.54	85.85 ± 20.16	<0.001
Albumin (g/L)	39.68 ± 3.23	28.26 ± 5.68	<0.001
Scr (mg/dL)	0.92 ± 0.19	1.68 ± 0.22	<0.001
SOFA score	-	5.02 ± 1.34	-
APACHE II score	-	12.02 ± 5.58	-

Note: BMI, body mass index; PCT, procalcitonin; WBC, white blood cell; CRP, C-reactive protein; Scr, serum creatinine; SOFA, sequential organ failure assessment; APACHE, acute physiology and chronic health evaluation.

### Expression of miR-381-3p and its clinical accuracy

**Figure 1.**
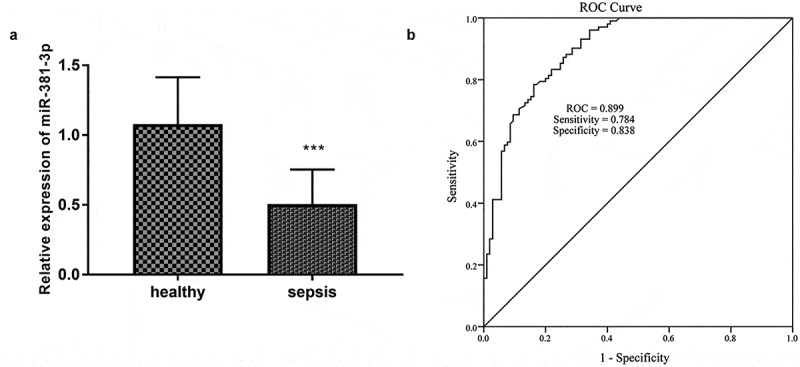
A total of 105 healthy controls and 102 sepsis patients were included in this study. (a) The expression miR-381-3p in sepsis. (b) The ROC curve showed the diagnosis importance of miR-381-3p. * **P < 0.001

As established in Figure 1(a), the expression of miR-381-3p was significantly decreased in the 102 sepsis patients (*P* < 0.001), providing that miR-381-3p might be associated with sepsis. The diagnostic value of miR-381-3p levels in sepsis was analyzed by the ROC curve, and the area under the curve (AUC) was o.899 (Figure 1(b)). When the cutoff value of miR-381-3p was 0.699, the sensitivity and specificity for the diagnosis of sepsis were 78.4% and 83.8% respectively (Figure 1(b)).

### The association between miR-381-3p and significant clinical characters

Furthermore, Pearson correlation was conducted in 102 sepsis patients and the consequence was expressed in Table 2. As exhibited, there were no correlations between miR-381-3p and age, body-mass index (BMI), gender, or albumin (*P* > 0.05). Interestingly, miR-381-3p was closely relative to the PCT (R = −0.878, *P* < 0.001), WBC (R = −0.810, *P* < 0.001), CRP (R = −0.821, *P* < 0.001), Scr (R = −0.668, *P* < 0.001), SOFA (R = −0.852, *P* < 0.001), and APACHE II (R = −0.725, *P* < 0.001).

**Table 2.** Correlation between the levels of miR-381-3p and clinical characteristics in 102 patients with sepsis

Parameters	The expression of miR-381-3p	
	*P* value	Correlation coefficient (*r*)
Age	0.969	0.004
BMI	0.983	0.002
Gender	0.961	−0.005
PCT	<0.001	−0.878
WBC	<0.001	−0.810
CRP	<0.001	−0.821
Albumin	0.085	0.117
Scr	<0.001	−0.668
SOFA score	<0.001	−0.852
APACHE II score	<0.001	−0.725

Note: BMI, body mass index; PCT, procalcitonin; WBC, white blood cell; CRP, C-reactive protein; Scr, serum creatinine; SOFA, sequential organ failure assessment; APACHE, acute physiology and chronic health evaluation.

### miR-381-3p protected H9c2 cells against sepsis injury

**Figure 2.**
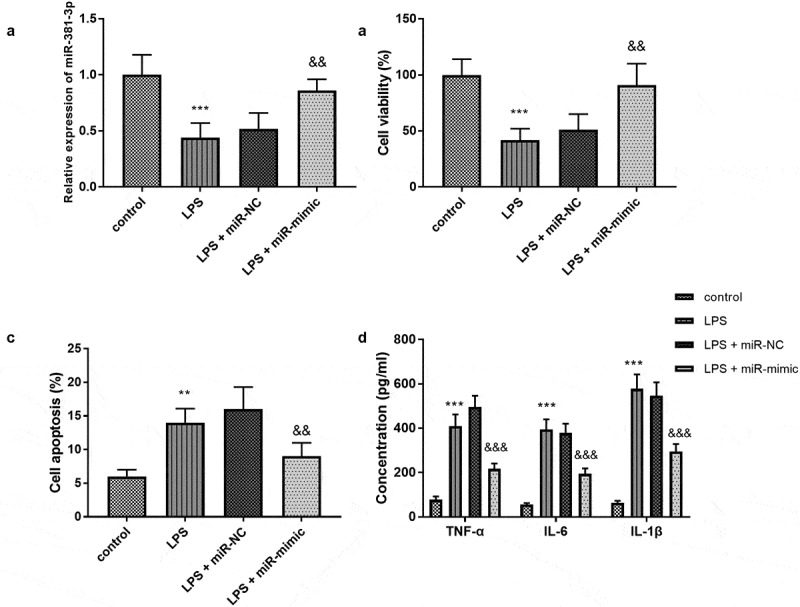
(a) MiR-381-3p mimics reversed the declined expression of miR-381-3p in H9c2 cells. The finding of (b) cell viability, (c) apoptosis, and (d) inflammation in vitro assay. ***P < 0.001, **P < 0.01, compared to control group; &&& *P* < 0.001, &&*P* < 0.01, compared to the LPS group

All experiments in H9c2 cells were repeated five times. In consideration of the abnormal expression of miR-381-3p in sepsis, the cell models were established and the levels of miR-381-3p were regulated artificially. The concentration of miR-381-3p was declined in the LPS group and elevated in the LPS + miR-mimic group (Figure 2(a), *p* < 0.01). Compared with the control group, the cell viability of the LPS group diminished significantly, the apoptosis rate significantly increased (Figure 2(b-c), *P* < 0.01). However, compared with the LPS group, the cell viability of the group increased significantly and the apoptosis rate decreased significantly (Figure 2(b-c), *P* < 0.01), indicating overexpression of miR-381-3p might play a protective role in the sepsis. In addition, the inflammatory activities were accelerated by the LPS treatment and ameliorated by the upregulation of miR-381-3p (Figure 2(d), *p* < 0.001).

### Protective roles of miR-381-3p on cardiac damage in vivo

**Figure 3.**
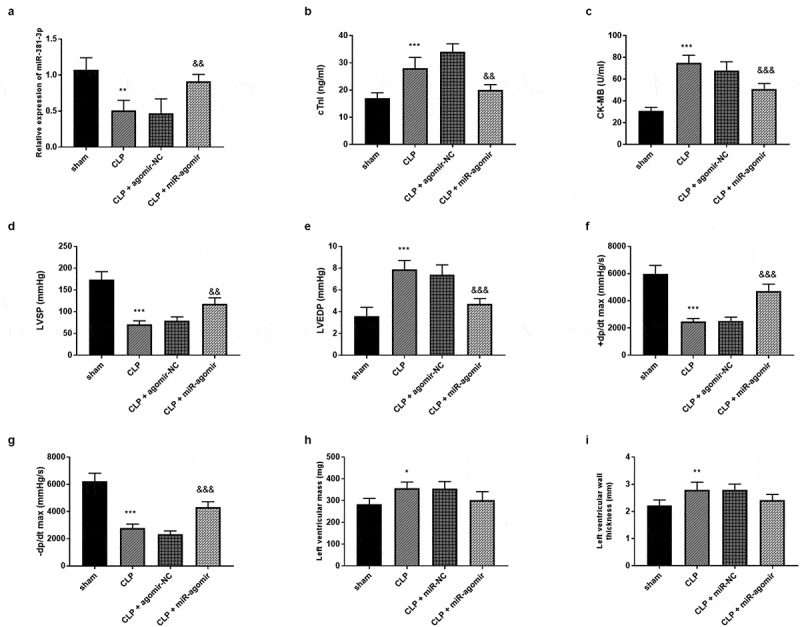
Each group included five rats. (a) MiR-381-3p mimics reversed the declined expression of miR-381-3p in SD rats. The finding of (b) CK-MB, (c) cTnI, (d) LVEDP, (e) LVSP, (f) +dp/dtmax, and (g)-dp/dtmax, (h) LVM, and (i) LVWT. ***P < 0.001, **P < 0.01, *P < 0.05, compared to sham group; &&& *P* < 0.001, && *P* < 0.01, compared to the CLP group

Each experiment was performed with five SD rats per group. As elucidated in Figure 3(a), there was a significant decrease of miR-381-3p expression in the CLP group, a significant increase in the CLP + miR-agomir group (*P* < 0.01). The secretion of the  cTnI were significantly raised in the CLP group relative to the sham group and had reduced tendencies in the CLP + miR-agomir group relative to the CLP group (Figure 3(b), *p* < 0.01). These findings clarified that miR-381-3p might protect SD rats against cardiac damage induced by sepsis. Additionally, the cardiac hemodynamic detection unveiled that miR-381-3p could repress the adverse influences of CLP on cardiac function by interfering the elevated CK-MB (Figure 3(c), *p* < 0.001), raising the LVSP values (Figure 3(d), *p* < 0.01), controlling the raised LVEDP (Figure 3(e), *p* < 0.001), and promoting ±dp/dtmax (Figure 3(f-g), *P* < 0.001). Besides, the LVM and LVWT in the CLP group were increased (Figure 3(h-I), *P* < 0.05), however, the miR-agomir transfection could not significantly change the LVM and LVWT of SD rat models (Figure 3(h–I), *P* > 0.05).

### Beneficial impacts of miR-381-3p on inflammation in vivo

**Figure 4.**
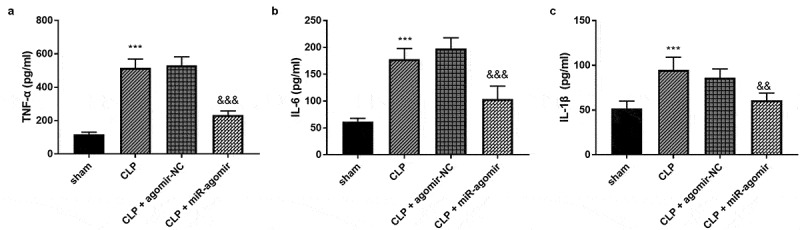
Each group included five rats. The alternations of (a) TNF-α, (b) IL-6, and (c) IL-1β. ***P < 0.001, compared to sham group; &&& *P* < 0.001, compared to the CLP group

Five rats were collected in each group. The tumor necrosis factor-alpha (TNF-α) accumulation was accelerated by the CLP surgery, while miR-381-3p changed the enhanced TNF-α levels (Figure 4(a), *p* < 0.001). Besides, the highly secreted interleukin-6 (IL-6) was steered in the CLP group, whereas, elevated miR-381-3p hindered the variation of IL-6 (Figure 4(b), *p* < 0.001). The abundance of interleukin-1beta (IL-1β) was also found in the CLP-elicited rats and raised IL-1β was qualified by the overexpression of miR-381-3p (Figure 4(c), *p* < 0.001).

### HMGB1 serves as a target gene of miR-381-3p

**Figure 5.**
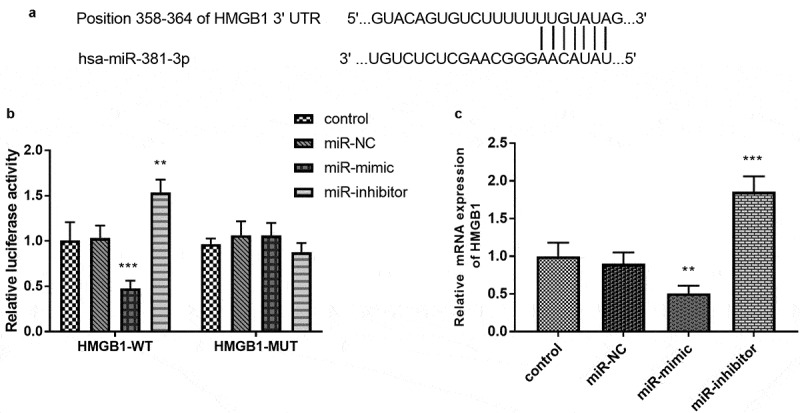
(a) The complementary sites between miR-381-3p and HMGB1. (b) The establishment of luciferase results. (c) overexpression of miR-381-3p restricted the mRNA expression of HMGB1, which was facilitated by the absence of miR-381-3p. ***P < 0.001, **P < 0.01

In Figure 5(a), the complementary bases were exhibited to reveal a possibility that HMGB1 could act as a target of miR-381-3p. The luciferase activity of the HMGB1-WT group showed that miR-381-3p mimics reduced the luciferase activity and miR-381-3p inhibitors improved the luciferase levels (Figure 5(b), *p* < 0.01). The results in Figure 5(c) demonstrated that upregulation of miR-381-3p attenuated the mRNA expression of HMGB1 and downregulation of miR-381-3p exerted a reversed function (*P* < 0.01).

## Discussion

Sepsis is a dangerous condition. If early diagnosis and treatment are not done in time, severe complications such as multiple organ dysfunction or even multiple organ failure will threaten the life safety of patients [18,19]. The heart is not only one of the main target organs of sepsis, but also an important effector organ of sepsis [20]. Researches have shown that about 64% of patients with sepsis have a sepsis-related myocardial injury [21]. Inflammatory cytokines play an important role in sepsis, which inhibits myocardial contractile function and leads to heart dysfunction [22]. Studies have also pointed out that the fatality rate of sepsis patients with myocardial injury is higher than that of sepsis patients alone [23]. Therefore, it is of great clinical significance to find new and effective diagnostic indicators related to sepsis.

A great deal of research has been done on the alternation of miRNAs in sepsis and their clinical significance. For instance, increased miR-155 levels are found in sepsis patients with acute lung injury relative to sepsis patients, and a high possibility of miR-155 predicting patients with sepsis and acute lung injury is also identified [24]. Besides, miR-328 is highly expressed in sepsis patients and rat models and it can distinguish sepsis patients from the healthy population [25]. In the present experiment, we verified a declined expression of miR-381-3p in patients with sepsis, elucidating that miR-381-3p might exert critical effects in progressive sepsis. Moreover, miR-381-3p showed higher accuracy, good specificity, and sensitivity in forecasting sepsis patients with promising. Importantly, close associations between miR-381-3p and clinical parameters of sepsis patients (such as PCT, WBC, CRP, Scr, SOFA, and APACHE II) were clarified, in order to further propose the underlying possibility that miR-381-3p regulated the development of sepsis. Our finding of qRT-PCR and further statistical analysis explain that the reduced miR-381-3p expression was a potential risk for sepsis patients.

MiRNA regulates the sequence of many genes in humans and plays an important role in the life process of humans [26]. In a report of 2020, upregulation of miR-223 can alleviate sepsis severity of septic cell models by inhibiting the abundance of pro-inflammatory indicators [27]. A report about miR-150-5p suggests that transfection of miR-150-5p mimics into H9c2 cells contributes to the decline in apoptosis [28]. In our experiment, the high concentration of miR-381-3p of septic H9c2 cells mitigated the damage of LPS to cell apoptosis and viability, indicating that miR-381-3p was a protective indicator in sepsis. Upregulation of miR-381-3p also repressed the inflammation irritated by LPS in cell models. In addition, these conclusions were further confirmed by in vivo experiments. We clarified the cardioprotective roles of miR-381-3p by the findings that augment of miR-381-3p expression moderated the injury of CLP on CK-MB, cTnI, and cardiac hemodynamic indexes. Besides, the enhancement of TNF-α, IL-6, and IL-1β in CLP-engendered was restored by the injection of miR-381-3p mimics. In ischemic stroke and cardiovascular diseases, overexpression of miR-381-3p moderates the inflammatory reactions [29,30]. In spinal cord injury, the enrichment of miR-381-3p may hinder the adverse effects of acute spinal cord injury by expanding the impacts on motor ability and inflammation [31]. Besides, Wang et al. study the influence of miR-378a-3p in CLP-treated rats, however, in the present study, we investigated the influence of miR-381-3p both in rat models and cell models of sepsis and further predicted the target gene of miR-381-3p [32].

Currently, the influence of aberrant HMGB1 expression in sepsis has been explored. It is reported that HMGB1 could mediate the treatment of sesamin on sepsis by modulating the pro-inflammatory cytokines [33]. Besides, high serum levels of HMGB1 are associated with death, and tissue damage in sepsis patients [34]. Our investigation examined and discovered that HMGB1 might be a downstream signaling component of miR-381-3p in sepsis by the luciferase results. In the meanwhile, the mRNA expression of HMGB1 was negatively accommodated by the variation of miR-381-3p levels. An observation from Zhu et al. substantiates that silenced miR-381-3p boosts the inflammation and apoptosis of vascular smooth muscle cells by competitively binding HMGB1 [30]. Kido et al. provide that cardiomyocytes’ apoptosis was decreased in the heart tissue with HMGB1 fragment, which indicates that HMGB1 may regulate the apoptosis of cardiomyocytes [35]. Another study suggests that HMGB1 can regulate the pro-inflammatory process and proliferation of cardiomyocytes [36]. All these investigations provide HMGB1 influences the phenotype of cardiomyocytes. Several limitations exist in this study, for instance, lack of further investigation of HMGB1, lack of some clinical data, and a retrospective design.

## Conclusion

Collectively, we found that the expression of miR-381-3p was significantly lessened in sepsis patients, LPS-steered cell models, and CLP-elicited rat models. The expression of miR-381-3p could serve as a biomarker for discriminating sepsis patients. The relationships between miR-381-3p and clinicopathological characters were also revealed. Furthermore, in vitro, miR-381-3p promoted cell viability, repressed cell apoptosis, and restricted cell inflammation. In vivo, miR-381-3p protected septic rats against myocardial injury and inflammation. HMGB1 was a promising target of miR-381-3p.

## Highlights

- The expression of miR-381-3p was declined in patients with sepsis.
  Enforced miR-381-3p expression restored the LPS-injured H9c2 cells.
- This study will be the first RCT to rigorously assess the efficacy and tolerability of the Rewind Technique.
- MiR-381-3p was beneficial to inflammatory and myocardial recovery.
- HMGB1 was a direct target of miR-381-3p.
